# The Impact of Sleep Loss on Screen Time in Children: Secondary Analyses of a Randomised Crossover Trial Using Objective Measures of Screen Time

**DOI:** 10.1111/ijpo.70050

**Published:** 2025-08-28

**Authors:** R. F. Jackson, K. A. Meredith‐Jones, J. J. Haszard, B. C. Galland, S. Morrison, M. Jaques, R. W. Taylor

**Affiliations:** ^1^ Department of Medicine University of Otago Dunedin New Zealand; ^2^ Haszard Biostatistics Kaka Point New Zealand; ^3^ Department of Pediatrics and Child Health University of Otago Dunedin New Zealand

**Keywords:** objective measures, randomised crossover trial., screen time, sleep, wearable cameras

## Abstract

**Background:**

How reduced sleep impacts screen time in children is unclear.

**Objectives:**

To explore how reduced sleep impacts objectively measured screen use.

**Methods:**

One hundred and five children (8–12 years) with caregiver‐reported sleep of 8–11 h/night were randomised to 7 nights sleep extension (go to bed 1 h earlier) or sleep restriction (bed 1 h later) in a crossover trial with a 7‐night washout between conditions. Sleep and time awake were measured using waist‐worn accelerometry (ActiGraph wGT3X‐BT) and screen time using wearable cameras (Brinno TLC130 Timelapse) and questionnaires. Camera images were coded as time spent on screens (raw data), including imputation for blocked images (Rules 1 and 2). Within‐person differences (95% CI) were calculated in those with matched camera data across sleep intervention weeks, in minutes and as percentage of awake time.

**Results:**

Screen time before school or on weekends did not differ in the 49 children (10.4 years, 51% female, 41% overweight, 78% European) with suitable camera data. After school, children appeared to have similar screen time using raw data (median difference; 25th, 75th percentiles: 18.7 min; −10.2, 72.5), but greater screen time during sleep restriction compared with extension after allowance for blocked images (Rule 2: 66.3 min; 7.5, 102.9 or 6% of awake time; 0.5, 10.0). Parents (*n* = 98) reported greater total screen use in children during the sleep restriction week (mean difference; 95% CI: 16.8 min; 1.8, 31.8).

**Conclusions:**

In this secondary analysis, getting less sleep appeared to increase screen time in children during the weekday afternoon and evening hours, compared to when they received more sleep.

**Trial Registration:** ACTRN12618001671257 Australian New Zealand Clinical Trials Registry; ANZCTR.

## 1 Introduction

Short sleep duration has been established as an independent risk factor for obesity in children [1, 2], raising questions regarding the effectiveness of sleep as a feasible strategy to prevent or treat childhood obesity. While the exact mechanisms remain unclear [3], the potential role of digital devices has come under scrutiny given increasing interest about the effects of excess screen use on child health [4, 5]. To date, the majority of literature has concentrated on how screens impact sleep [6, 7, 8], with relatively few studies examining whether a lack of sleep impacts screen use behaviour in children. A handful of longitudinal studies have shown that the relationship between sleep and screen use may be bi‐directional [9, 10, 11], but only one intervention trial appears to have explored how reduced sleep influences how children use their screens. The mechanistic study of Hart et al. [12] showed that a reduction in sleep of more than 2 h per night led to an additional hour of television each day. However, data were parent‐reported and other device use was not measured, likely leading to substantial underestimation of total screen time [13].

To date, most screen‐based research has been limited to the use of subjective, predominantly questionnaire‐based measures of screen use [14], which are reliant on memory and may be subject to social desirability bias, making it hard to determine true relationships between screen use and health [15]. More recently, wearable cameras have been tested as a viable option to objectively capture screen use in youth [16, 17, 18]. However, they do not appear to have been used to objectively measure screen use within an intervention study, particularly in the context of how sleep may impact screen use in children.

Considering the limited mechanistic evidence investigating the relationship between sleep and obesity [12, 19, 20], we recently undertook a large randomised cross‐over trial (DREAM study) to determine what happens to energy‐balance behaviours when children get less sleep [21]. We showed that when children sleep less, they eat more, particularly highly processed, non‐core foods [22]. They were also more sedentary as measured using accelerometry [23], but accelerometers are unable to contextualise what type of sedentary behaviours are being undertaken. Therefore, the aim of this secondary analysis was to explore how getting less sleep, compared to getting more sleep, impacts children's screen use behaviour, using objective measures of screen use. We hypothesised that their screen time would increase when they were more likely to be tired during sleep restriction.

## 2 Methods

The daily rest, eating, activity and monitoring (DREAM) study was a randomised crossover trial examining the effect of mild sleep deprivation on eating behaviour and activity patterns in children (approved by the University of Otago Human Ethics Committee, Reference #18/146). As protocol [21] and main outcome [24] papers are available, only relevant methods will be discussed briefly here. Healthy children aged 8–12 years were recruited from Dunedin, New Zealand (October 2018–March 2020) and were eligible if their caregiver reported they slept 8–11 h per night and had no diagnosed sleep disorders. After obtaining written consent (caregiver) and assent (child), baseline measurements of usual sleep were obtained over 7 nights. Recruitment finished slightly short of target (*n* = 110 participants) due to COVID‐19 restrictions, but dropout was considerably less than anticipated [21]. Randomisation was generated by the study biostatistician, using random block lengths (Stata 15.1, StataCorp, Texas) in a 1:1 allocation, stratified by age (8–10, 11–12 years) and gender, and uploaded to the research management program (REDCap) randomisation module. Participants were randomised complete either 7 nights of sleep restriction (go to bed 1 h later than baseline usual) or 7 nights of sleep extension (go to bed 1 h earlier than baseline usual). Wake times were kept consistent, and a 7‐night washout period between intervention weeks ensured sleep could return to normal before completion of the alternate intervention.

Demographic characteristics collected at baseline included the child's age, sex, and ethnicity as well as maternal education level. Socio‐economic status was assessed using the New Zealand Index of Deprivation 2018, which uses nine variables from the New Zealand census to provide a composite measure of area‐level deprivation based on the participant's home address, which is then split by deciles from 1 (least deprived) to 10 (most deprived) [25]. Anthropometric data were obtained at baseline by trained researchers with participants wearing light clothing and no shoes. Height was measured to the nearest 0.5 cm using a portable stadiometer (Wedderburn Portable Height Rod, WS‐HR) and weight to the nearest 0.1 kg using portable electronic scales (Tanita electronic scales HD351) in duplicate using standard procedures. Body Mass Index (BMI) z‐scores were calculated and categorised based on the WHO growth reference for 5–19 year olds [26]; with normal weight defined as > −2SD to +1SD, overweight as > +1SD to +2SD, and obese as > +2SD.

Screen use was measured objectively through wearable camera images, captured at 2 s intervals. Screen use was added as a further secondary outcome after initial trial registration. Participants were asked to wear the Brinno TLC130 Timelapse camera (Brinno, Taipei City, Taiwan) on a harness around the chest for 2 days (Day 3 and Day 7) of each intervention week, from the time they woke up to the time they went to bed. Cameras faced outwards and captured screen use. Images were downloaded onto the University of Otago's high‐capacity storage and later coded using Timelapse 2.0 (University of Calgary, Canada), for the type of screen (television, mobile, tablet, gaming console, computer/laptop/other), screen behaviour (regulated watching [e.g., Netflix and free‐to‐air television], unregulated watching [e.g., YouTube and TikTok videos], watching unknown, gaming/watching gaming, browsing/social media, communication, educational/creative, other), priority (screens which the child appears actively engaged with) versus background (screens that the child does not appear engaged with) screens, multi‐screen use (use of more than one device simultaneously), and any blocked images (e.g., by clothing, bedcovers, positioning of the child). Two researchers coded 100% of the images, with the head coder (RJ) visually checking all image sets for completion. For each participant, one coder was responsible for coding all time‐blocks for both sleep extension and sleep restriction, to ensure consistency within participants. Further information is available online [27].

Participants were included in this analysis if they had valid wear time for the before school, after school, or weekend time blocks, for at least one matched day for sleep extension and sleep restriction weeks. For wear time to be considered valid, each participant had to wear the camera on the same day of each week in sleep restriction and sleep extension conditions; for example, if the camera was worn on school days, the participant needed to attend school on both days to be included. Participants who were home schooled were only included if they had valid weekend days, as they indicated that they usually did not have set ‘school times’, making it difficult to classify before and after school. The awake window was calculated from the time the participant woke up (measured using accelerometry) to the prescribed bedtime for that night, rather than from sleep onset since this was a sleep manipulation protocol. Valid wear time was defined as: before school = at least 60 min of wear time; after school = no more than 2 h of their awake window missing; weekend = no more than 4 h of their awake window missing.

**FIGURE 1:**
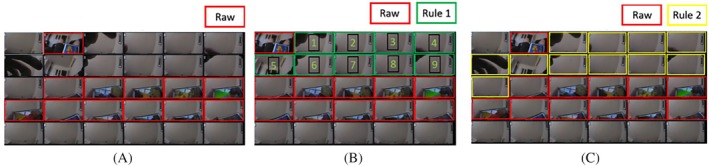
Example of coding rules. (A) Raw data where only images with screens are included in screen time estimates. (B) Rule 1 where raw data and up to 10 consecutive no‐screen images (i.e., up to 20 s with a screen out of view) as long the priority device and activity on either side of the 10 images were the same. (C) Rule w which included raw data and an unlimited number of consecutive no‐screen images as long the priority device and activity on either side of the images were the same. Note for this set of images, rule 1 would not apply as there are more than 10 consecutive images between the episodes of images with screens (raw data highlighted in red).

Missing or blocked camera images were labelled as such during the coding process. Assumptions about the number of blocked images allowed to still be classified as screen time were handled post‐coding rather than as part of the coding process that has been done previously [28]. Three different coding rules were used to estimate screen use in our different categories of interest. Screen use was calculated using (1) raw data where only those images that clearly contained screens were summed to calculate screen use; (2) rule 1—a conservative assumption that included raw data and up to 10 consecutive no‐screen images (i.e., up to 20 s with a screen out of view) as long as the priority device and activity on either side of the 10 images were the same; rule 2—similar to rule 1 but less conservative, with unlimited consecutive images that contained a blocked code, reclassified to screen images if there was an image on either side of the blocked episode that had the same priority device and activity code. Figure 1 illustrates an example of screen images dipping in and out of view using these rules.

Screen use was also determined by parental report using the screen‐based questions from the Youth Activity Profile (YAP) [29]. Parents were asked to indicate how much time their child spent watching five different devices types (television, video games, computers, phones and tablets) outside of school time on (i) weekdays and (ii) weekends over the past 7 days. Five answer options were provided for each device: ‘not really used at all’, ‘used less than 1 hour per day’, ‘used for 1‐2 hours per day’, ‘used for 2 to 3 hours per day’, and ‘used for more than 3 hours per day’. Data were scored as 0, 0.5, 1.5, 2.5, 3.5, weighted for weekend/weekday, and summed to give average usual screen time per day.

Total sleep time was measured during baseline and each intervention week using accelerometers (ActiGraph wGT3X‐BT, Pensacola, USA) worn at the hip 24‐h a day for 7 days. ActiGraphs were initialized using ActiLife software (version 9.0.0), and data were cleaned and scored with an automated script developed in MatLab (MathWorks, Natick, Massachusetts) [30], which provides valid estimates of sleep in this age group [31]. Total sleep time refers to the time between sleep onset and offset, minus any time awake in the night. A day was considered valid if the child did not remove the Actigraph during the sleep period and wore the device for ≥ 8 h with < 2 h of non‐wear time while awake. Total awake time was calculated as the accelerometery derived total time spent awake including total non‐wear time for participants who had valid data. Data were averaged across the week, weighted for weekend days (2/7) and weekdays (5/7), and normalised to 24 h [32]. An a priori per protocol definition was set as a mean difference in total sleep time of at least 30 min between intervention weeks [21].

Descriptive statistics are presented to explore how mild sleep deprivation may affect screen use within time blocks (before school, after school and weekend) to represent non‐school based screen use. Mean and standard deviations for each screen use measure within the sleep extension week and sleep restriction week were calculated. If participants had valid data for two before school or after school time blocks per week, then the average of the two for each screen use measurement was calculated. The within‐person differences between the sleep restriction and extension weeks were then calculated for each participant, and the median value with 25th and 75th percentiles presented for each screen use measure as an absolute total and as a percentage of their awake time. As this was an exploratory analysis, no significance testing was undertaken. A sample size calculation was undertaken for the primary outcome [21], but not for these analyses.

## 3 Results

**FIGURE 2:**
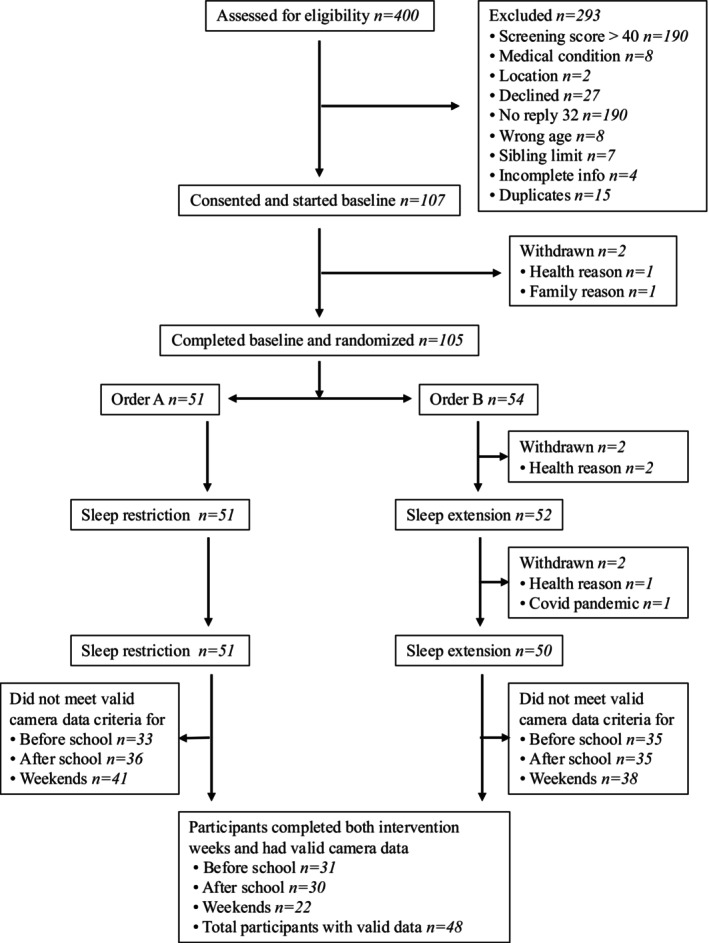
Consort flow diagram of DREAM participants included in the screens analysis.

Figure 2 presents the consort diagram for participants included in the DREAM screen use analysis. Briefly, 400 children were screened, of which 105 participated in the full trial. The final samples for these analyses included 49 children with valid matching camera data for at least one of the time blocks (morning, after school, evening) and 98 children with questionnaire measures during both intervention weeks. Table 1 demonstrates that baseline characteristics for participants included in the camera analysis were similar to the full sample, although a higher percentage of these participants (74.5%) met the per protocol average sleep difference of at least 30 min per night. The average age of camera participants was 10.4 years (SD 1.4) with 40.9% of the sample classified within the overweight/obese category. The sample mostly identified as New Zealand European, with 14.3% of the sample identifying as Māori (indigenous population of New Zealand). The questionnaire sample were very similar to the full sample.

**TABLE 1:** Baseline demographics.

Variable		Full DREAM sample *n* (%)	Sample who met camera criteria *n* (%)^a^, ^h^	Sample who met questionnaire criteria *n* (%)
*n*		105	49	98
Gender	Male	52 (49.5)	24 (49.0)	46 (46.9)
	Female	53 (50.5)	25 (51.0)	52 (53.1)
Age, years mean (SD)		10.3 (1.4)	10.4 (1.4)	10.3 (1.4)
Weight status^b^, ^d^	Normal weight	64 (61.0)	29 (59.1)	60 (61.2)
	Overweight	24 (22.9)	13 (26.5)	23 (23.5)
	Obese	16 (16.0)	7 (14.3)	15 (15.3)
Ethnicity	European/Other	82 (78.1)	38 (77.6)	76 (77.6)
	Māori	16 (15.2)	7 (14.3)	15 (15.3)
	Pacific	3 (2.9)	2 (4.1)	3 (3.1)
	Asian	4 (3.8)	2 (4.1)	4 (4.1)
Area‐level deprivation^c^	High (NZDep 8–10)	22 (21.0)	8 (16.3)	20 (20.4)
	Medium	44 (41.9)	20 (40.8)	39 (39.8)
	Low (NZDep 1–3)	39 (37.1)	21 (42.9)	39 (39.8)
Maternal education level	Secondary school only	25 (23.8)	11 (22.5)	22 (22.5)
	Postsecondary	28 (26.7)	20 (40.8)	25 (26.6)
	University degree or higher	48 (45.7)	18 (36.7)	47 (50.0)
Total who met per protocol^d^		59 (56.2)^f^	35 (74.5)^g^	59 (60.0)
Total sleep time difference (SD) in minutes^e^		36.8 (43.4)	42.7 (45.5)	37.1 (44.0)

^a^ Participants who had valid camera data in both extension and restriction weeks for either before school, after school, or weekend data.

^b^ Using WHO reference data [26].

^c^ Uses the New Zealand Index of Deprivation 2018 [25] which reflects the extent of material and social deprivation and is used to construct deciles from 1 to 10 (least deprived) to 10 (most deprived).

^d^ Difference between sleep onset and sleep offset minus any time awake in the night.

^e^ Prespecified sleep difference was 30 min less in the restriction week compared to the extension week. Participants included in the ‘per protocol’ analyses were required to have ≥ 3 valid days.

^f^ 96 participants had valid accelerometry data for this analysis.

^g^ 47 participants had valid accelerometry data for this analysis.

^h^ *n* = 6 participants had valid data at all timepoints, *n* = 17 with valid data for weekday before school and after school, *n* = 10 with valid data for weekday after school and weekends, *n* = 11 with valid data for weekday before school and weekends, *n* = 7 with valid data for weekends only, *n* = 6 with valid data for weekday after school only, *n* = 9 with valid data for weekday before school only, *n* = 22 with valid data for only one session (weekend OR before school OR after school).

Table 2 shows the mean (SD) screen use outcomes for sleep extension and sleep restriction and the median difference between the intervention weeks, by time blocks. During the week of sleep restriction, participants wore the camera considerably more during the after‐school time block (median difference 101.9 min) and weekend time block (median difference 121.6 min), but not in the before school session (median difference −0.7 min). In participants who had valid data for the before school session (*n* = 31), median differences in screen use when using any coding rules were very small, all less than 2 min. In those with valid data for the after‐school time block (*n* = 30), participants appeared to have greater total screen use (median difference 18.7 min) during sleep restriction, a difference that was heightened when using the rule 2 coding assumption (median difference 66.3 min). Patterns were similar for priority screen use but not background or multiple screen use. Participants with valid data for the weekend time block (*n* = 22) generally used screens less in the week of sleep restriction, but median differences were all less than 14 min.

**TABLE 2:** Differences in screen use (minutes) between sleep extension and sleep restriction by time block (before school, after school and weekend), in participants who met the valid time criteria.

Time block^a^	Screen measure^b^	Rule^c^	Sleep extension (min) mean (SD)	Sleep restriction (min) mean (SD)	Median (25th, 75th) difference (min)
Before school (*n* = 31)	Total valid time		92.4 (34.9)	93.9 (30.1)	−0.7 (−13.4, 20.0)
	Total screen use	Raw	18.2 (25.2)	16.1 (17.0)	1.2 (−7.8, 8.9)
		Rule 1	19.0 (25.9)	17.0 (17.8)	1.2 (−6.8, 9.2)
		Rule 2	22.5 (28.6)	20.8 (21.5)	1.2 (−10.0, 10.9)
	Priority screen use	Raw	14.8 (23.5)	12.9 (17.4)	0.0 (−4.8, 6.0)
		Rule 1	15.6 (24.2)	13.8 (18.3)	0.0 (−4.9, 6.1)
		Rule 2	19.4 (27.0)	17.7 (21.9)	0.0 (−12.9, 6.2)
	Background screen use		3.4 (5.3)	3.2 (4.2)	0.0 (−0.4, 1.3)
	Multiple screen use		1.6 (4.7)	0.4 (1.5)	0.0 (0.0, 0.0)
After school (*n* = 30)	Total valid time		240.1 (48.2)	338.0 (61.5)	101.9 (83.0, 126.6)
	Total screen use	Raw	64.3 (49.0)	92.7 (58.4)	18.7 (−10.2, 74.6)
		Rule 1	68.1 (50.9)	96.5 (59.5)	21.0 (−13.7, 79.2)
		Rule 2	99.7 (68.4)	147.2 (81.1)	66.3 (7.5, 102.9)
	Priority screen use	Raw	56.8 (49.4)	83.0 (55.7)	24.7 (−4.6, 72.9)
		Rule 1	60.8 (51.8)	86.9 (56.9)	26.7 (−6.2, 76.0)
		Rule 2	93.6 (69.0)	138.4 (84.1)	59.0 (8.0, 104.9)
	Background screen use		7.6 (6.5)	9.8 (9.3)	0.4 (−2.4, 4.0)
	Multiple screen use		7.7 (16.9)	9.7 (18.6)	0.1 (−0.4, 9.3)
Weekend (*n* = 22)	Total valid time		643.3 (75.3)	753.1 (67.8)	121.6 (71.2, 143.0)
	Total screen use	Raw	212.7 (122.6)	216.7 (145.6)	−12.6 (−103.7, 80.4)
		Rule 1	222.4 (125.9)	227.2 (149.4)	−13.9 (−114.4, 85.0)
		Rule 2	276.9 (136.3)	289.1 (159.1)	−0.2 (−118.7, 131.1)
	Priority screen use	Raw	186.8 (116.0)	190.7 (140.2)	−0.7 (−100.7, 72.9)
		Rule 1	197.7 (120.0)	201.9 (144.5)	−3.4 (−109.0, 77.3)
		Rule 2	254.4 (131.6)	267.8 (160.1)	−11.0 (−110.2, 130.8)
	Background screen use		25.9 (21.6)	25.9 (21.8)	−1.5 (−16.2, 7.9)
	Multiple screen use		28.5 (40.8)	37.8 (70.3)	0.4 (−9.3, 32.4)

^a^ Before school was the time from when the participant woke up until 9 am on a school day; After school was the time from 3 pm to the time the participant went to sleep on a school day; weekends was from the time the participant woke up until they went to sleep.

^b^ Priority screen use refers to when the participant was actively engaged with a visible device; background screen use refers to when a screen is visible but there was no engagement; multiple screen use refers to when at least two screens were visible.

^c^ Raw data were calculated from the sum of all images that contained a screen with no assumptions made. Rule 1 included periods where there were no screens as long as this was for 20 s or less (i.e., ≤ 10 images) and there was the same screen time code on either side of the “no screen” episode. Rule 2 included all consecutive images that were coded as blocked or uncodable if there was an image before and after that episode that was coded for the same device and activity (priority screens only).

Table 3 shows differences in screen use, presented as a percentage of wake time during the day given that children had more opportunity to use screens when sleep was restricted as their wake time was longer. In other words, this analysis was undertaken to determine whether any differences in screen use observed occurred over and above the extra time available to use screens when able to stay up later. Participants who had valid data for the after school time block and weekend time blocks appeared to wear the camera for a higher percentage of their awake time during sleep restriction (after school 9.2%, weekend 9.9%). However, with regards to screen use outcomes, meaningful differences were only observed in the after‐school time block, with these participants appearing to use screens more, even after effectively adjusting for their additional awake time, during the week of sleep restriction (e.g., rule 2 median difference 6% (25th, 75th percentiles: 0.5, 10.0)).

**TABLE 3:** Differences in screen use (% of time awake) between sleep extension and sleep restriction by time block (before school, after school and weekend), in participants who met the valid time criteria.

Time block^a^	Screen measure^b^	Rule^c^	Sleep extension (%) mean (SD)	Sleep restriction (%) mean (SD)	Median (25th, 75th) difference (%)
Before school (*n* = 29)^d^	Total valid time		10.9 (3.9)	10.5 (3.3)	−0.2 (−2.1, 1.7)
	Total screen use	Raw	2.2 (2.9)	1.8 (1.9)	0.0 (−0.8, 0.9)
		Rule 1	2.3 (3.0)	1.9 (2.0)	0.0 (−0.6, 1.2)
		Rule 2	2.7 (3.3)	2.4 (2.4)	0.0 (−1.3, 1.1)
	Priority screen use	Raw	1.8 (2.7)	1.5 (2.0)	0 (−0.6, 0.6)
		Rule 1	1.9 (2.8)	1.6 (2.1)	0 (−0.7, 0.6)
		Rule 2	2.3 (3.1)	2.0 (2.4)	0 (−1.3, 0.6)
After school (*n* = 28)^d^	Total valid time		27.5 (5.1)	36.5 (6.6)	9.2 (5.8, 12.8)
	Total screen use	Raw	7.6 (5.7)	9.9 (6.6)	1.6 (−1.5, 7.8)
		Rule 1	8.0 (5.9)	10.3 (6.7)	1.8 (−1.9, 8.2)
		Rule 2	11.6 (7.9)	15.6 (8.9)	6.0 (0.5, 10.0)
	Priority screen use	Raw	6.7 (5.7)	8.9 (6.3)	2.0 (−1.3, 7.2)
		Rule 1	7.2 (6.0)	9.3 (6.4)	2.0 (−1.8, 7.3)
		Rule 2	11.0 (8.0)	14.7 (8.8)	5.5 (0.2, 8.7)
Weekend (*n* = 21)^d^	Total valid time		75.4 (7.8)	84.0 (7.4)	9.9 (2.0, 15.8)
	Total screen use	Raw	25.0 (14.6)	24.8 (17.9)	0.8 (−12.7, 8.2)
		Rule 1	26.1 (15.0)	26.0 (18.4)	0.4 (−13.6, 8.6)
		Rule 2	32.8 (16.3)	32.2 (19.1)	−2.7 (−13.9, 11.0)
	Priority screen use	Raw	22.9 (13.8)	21.9 (17.2)	−0.6 (−11.9, 7.6)
		Rule 1	23.32 (14.3)	23.1 (17.8)	−0.6 (−12.8, 7.9)
		Rule 2	30.1 (15.8)	29.8 (19.2)	−2.6 (−13.1, 9.3)

^a^ Before school was the time from when the participant woke up until 9 am on a school day; after school was the time from 3 pm to when the participant went to sleep on a school day; weekends was from the time the participant woke up until they went to sleep.

^b^ Priority screen use refers to when the participant was actively engaged with a visible device.

^c^ Raw data were calculated from the sum of all images that contained a screen with no assumptions made. Rule 1 included periods where there were no screens as long as this was for 20 s or less (i.e., ≤ 10 images) and there was the same screen time code on either side of the ‘no screen’ episode. Rule 2 included all consecutive images that were coded as blocked or uncodable if there was an image before and after that episode that was coded for the same device and activity (priority screens only).

^d^ *n* refers to those with valid accelerometry data to determine total awake time.

Tables 4 and 5 report differences in screen use between intervention weeks by screen type and behaviour respectively. Results are presented as those who used the specific device or activity at any time during either week of intervention, and for priority screens only. Television was the most used device, with regulated watching (i.e., watching where the content is not published by the public including free to air television or Netflix shows), being the most common activity coded. While very small median differences were observed in the before school time block, participants appeared to engage with television more (median difference range from 14.8 using raw data to 42.9 min when using rule 2) in the after school block. Participants also appeared to engage in more regulated watching (median differences ranging from 6.9 min when using raw data to 23.7 min when using rule 2) during sleep restriction. For weekend days (all day data), participants appeared to have similar device use, with a small increase in mobile phone use (median differences ranging from 3.5 min when using raw data to 7.4 min when using rule 2). In participants who engaged in gaming or watching games, these participants appeared to game/watch gaming more on the weekends, while those who engaged in regulated watching also appeared to engage with this activity more (median difference ranging from 7.3 min when using raw data to 34.4 min when using rule 2).

**TABLE 4:** Differences in screen use by device type between sleep extension and restriction, by time block, in participants who engaged with the devices.

Time block^a^	Device^b^	Rule^c^	*N*	Sleep extension (min) mean (SD)	Sleep restriction (min) mean (SD)	Median (25th, 75th) difference (min)
Before school	Television	Raw	17	13.3 (14.9)	15.9 (19.3)	0.2 (−7.3, 12.2)
		Rule 1		14.0 (15.2)	16.9 (20.1)	0.4 (−8.1, 12.6)
		Rule 2		17.4 (15.8)	18.4 (21.7)	2.2 (−15.0, 13.2)
	Mobile phone	Raw	16	7.6 (15.9)	2.9 (4.6)	−0.3 (−8.7, 0.4)
		Rule 1		8.0 (16.4)	3.1 (4.8)	−0.4 (−9.9, 0.4)
		Rule 2		10.7 (19.1)	5.5 (9.2)	0.7 (−11.4, 0.7)
	iPad/Tablet	Raw	5	7.6 (15.9)	2.9 (4.6)	−0.3 (−8.7, 0.4)
		Rule 1		8.0 (16.4)	3.1 (4.8)	−0.4 (−9.9, 0.4)
		Rule 2		10.7 (19.1)	5.5 (9.2)	0.7 (−11.4, 0.7)
	Computer and other	Raw	15	7.6 (15.9)	2.9 (4.6)	−0.3 (−8.7, 0.4)
		Rule 1		8.0 (16.4)	3.1 (4.8)	−0.4 (−9.9, 0.4)
		Rule 2		10.7 (19.1)	5.5 (9.2)	0.7 (−11.4, 0.7)
After school	Television	Raw	25	23.4 (22.9)	49.1 (39.8)	14.8 (1.3, 48.6)
		Rule 1		25.7 (24.9)	51.7 (40.4)	15.4 (0.3, 50.2)
		Rule 2		40.7 (39.3)	78.7 (50.2)	42.9 (3.8, 67.5)
	Mobile phone	Raw	24	12.2 (18.5)	9.3 (12.9)	−0.1 (−4.8, 1.8)
		Rule 1		13.0 (19.6)	10.3 (14.0)	−0.2 (−4.7, 2.4)
		Rule 2		25.1 (31.5)	27.9 (54.8)	0.9 (−20.5, 2.7)
	iPad/Tablet	Raw	14	21.2 (30.3)	15.5 (17.0)	2.0 (−20.9, 11.3)
		Rule 1		22.4 (31.9)	16.4 (18.2)	2.3 (−21.8, 11.8)
		Rule 2		37.2 (54.2)	42.1 (69.0)	10.9 (−31.5, 30.7)
	Computer and other	Raw	22	25.6 (35.4)	38.0 (43.4)	10.8 (−11.1, 42.4)
		Rule 1		26.8 (37.1)	38.9 (44.3)	10.0 (−11.5, 44.0)
		Rule 2		34.0 (39.3)	44.6 (47.9)	8.9 (−30.0, 59.4)
Weekends	Television	Raw	22	118.8 (100.6)	117.0 (105.2)	−0.6 (−65.0, 51.5)
		Rule 1		126.0 (104.0)	124.3 (109.3)	−3.3 (−68.4, 49.7)
		Rule 2		153.0 (107.8)	156.5 (118.6)	−9.3 (−54.5, 77.6)
	Mobile phone	Raw	17	14.1 (21.2)	24.5 (37.1)	3.5 (0.5, 18.0)
		Rule 1		15.3 (22.6)	26.0 (38.4)	3.5 (0.7, 18.0)
		Rule 2		27.4 (34.1)	48.1 (69.5)	7.4 (−3.0, 27.1)
	iPad/Tablet	Raw	12	54.7 (61.2)	32.0 (47.0)	−1.1 (−71.8, 0.8)
		Rule 1		56.9 (63.4)	33.6 (49.4)	1.1 (−74.2, 0.9)
		Rule 2		75.8 (86.4)	47.4 (77.5)	−0.1 (−77.0, 26.9)
	Other	Raw	16	39.8 (65.8)	67.5 (101.1)	2.2 (−14.5, 50.2)
		Rule 1		42.2 (70.6)	70.6 (103.5)	2.6 (−15.5, 51.8)
		Rule 2		56.7 (83.4)	89.8 (114.1)	3.2 (−17.7, 41.1)

^a^ Before school was the time from when the participant woke up until 9 am on a school day; after school was the time from 3 pm to the time the participant went to sleep on a school day; weekends was from the time the participant woke up until they went to sleep.

^b^ The other category included other devices such as gaming consoles, laptop computers, desktop computers, and digital watches.

^c^ Raw data were calculated from the sum of all images that contained a screen with no assumptions made. Rule 1 included periods where there were no screens as long as this was for 20 s or less (i.e., ≤ 10 images) and there was the same screen time code on either side of the “no screen” episode. Rule 2 included all consecutive images that were coded as blocked or uncodable if there was an image before and after that episode that was coded for the same device and activity (priority screens only).

**TABLE 5:** Differences in screen use by screen behaviour between sleep extension and restriction, by time block, in participants who engaged with the devices.

Time block^a^	Screen behaviour^b^	Rule^c^	*n*	Sleep extension (min) mean (SD)	Sleep restriction (min) mean (SD)	Median (25th, 75th) difference (min)
Before school	Regulated watching	Raw	18	10.0 (10.6)	9.7 (14.2)	0.0 (−7.0, 2.7)
		Rule 1		10.9 (11.1)	10.3 (14.9)	0.1 (−8.0, 3.3)
		Rule 2		14.6 (12.7)	12.4 (17.7)	−0.9 (−14.5, 3.6)
	Unregulated watching	Raw	11	6.4 (14.0)	3.4 (4.6)	0.1 (−3.7, 2.1)
		Rule 1		6.9 (15.0)	3.5 (4.6)	−0.2 (−3.7, 2.4)
		Rule 2		8.6 (17.5)	5.2 (5.9)	−1.1 (−3.7, 0.6)
	Watching unknown	Raw	12	0.6 (1.7)	0.2 (0.4)	0.0 (−0.3, 0.2)
		Rule 1		0.6 (1.7)	0.2 (0.4)	0 (−0.3, 0.4)
		Rule 2		0.8 (2.2)	0.3 (0.5)	−0.1 (−0.3, 0.5)
	Gaming/watching gaming	Raw	12	10.4 (16.9)	8.8 (17.3)	0.5 (−10.2, 2.5)
		Rule 1		10.9 (17.1)	9.5 (18.4)	0.5 (−11.1, 2.6)
		Rule 2		11.7 (17.6)	10.1 (18.6)	0.5 (−13.3, 2.6)
	Browsing/social media	Raw	20	1.4 (2.0)	1.3 (2.4)	−0.2 (−0.8, 0.9)
		Rule 1		1.5 (2.1)	1.6 (2.7)	0.2 (−0.9, 1.0)
		Rule 2		2.7 (3.8)	3.5 (6.3)	−0.2 (−1.5, 1.1)
	Communication	Raw	2	0.1 (0.1)	0.2 (0.2)	0.1 (−0.2, 0.3)
		Rule 1		0.1 (0.1)	0.2 (0.2)	0.1 (−0.2, 0.3)
		Rule 2		0.5 (0.7)	0.9 (1.12)	0.4 (−0.9, 1.7)
	Educational/creative	Raw	5	1.8 (2.6)	3.5 (4.7)	1.1 (−1.6, 5.2)
		Rule 1		1.9 (2.7)	4.5 (6.5)	1.1 (−1.6, 7.7)
		Rule 2		1.9 (2.8)	8.7 (14.7)	2.8 (−1.7, 17.2)
	Other	Raw	19	0.7 (1.4)	0.3 (0.5)	0.1 (−0.7, 0.1)
		Rule 1		0.9 (1.8)	0.4 (0.6)	−0.1 (−1.1, 0.1)
		Rule 2		1.8 (3.1)	0.9 (1.3)	−0.2 (−1.5, 0.2)
	Uncodable		20	1.2 (1.9)	1.4 (2.0)	0.0 (−1.1, 1.8)
After school	Regulated watching	Raw	28	17.8 (21.2)	32.8 (34.2)	6.9 (−4.9, 40.2)
		Rule 1		19.4 (23.0)	35.2 (36.3)	7.6 (−5.2, 44.0)
		Rule 2		30.8 (37.2)	56.6 (51.6)	23.7 (−3.3, 59.6)
	Unregulated watching	Raw	20	13.3 (17.6)	18.7 (18.8)	6.3 (−11.6, 25.0)
		Rule 1		14.8 (19.6)	20.3 (20.2)	7.0 (−12.7, 24.5)
		Rule 2		30.8 (40.5)	36.2 (33.5)	5.5 (−20.9, 36.6)
	Watching unknown	Raw	24	1.7 (2.1)	2.8 (3.8)	0.1 (−0.1, 1.3)
		Rule 1		2.1 (2.8)	3.1 (4.1)	0.1 (−0.1, 1.3)
		Rule 2		3.8 (6.5)	14.9 (21.8)	1.3 (−0.2, 16.7)
	Gaming/watching gaming	Raw	19	23.8 (41.7)	25.9 (37.5)	−0.1 (−4.3, 25.7)
		Rule 1		25.6 (44.6)	26.5 (38.1)	−0.1 (−4.9, 27.6)
		Rule 2		38.2 (51.0)	30.5 (42.1)	−1.5 (−30.7, 1.9)
	Browsing/social media	Raw	26	5.9 (7.4)	7.8 (11.2)	0.1 (−3.7, 8.7)
		Rule 1		6.4 (8.1)	8.4 (11.5)	0.3 (−4.5, 8.6)
		Rule 2		10.7 (12.3)	18.9 (21.7)	1.5 (−3.2, 20.6)
	Communication	Raw	6	1.8 (3.9)	0.2 (0.2)	0.1 (−3.1, 0.3)
		Rule 1		1.9 (4.2)	0.2 (0.2)	0.1 (−3.5, 0.3)
		Rule 2		2.4 (4.4)	2.2 (3.3)	0.2 (−5.4, 6.2)
	Educational/creative	Raw	9	4.3 (5.4)	10.4 (10.5)	2.9 (−3.0, 18.5)
		Rule 1		4.8 (6.1)	10.8 (11.0)	2.3 (−3.0, 19.3)
		Rule 2		7.5 (10.2)	15.9 (17.8)	4.3 (−9.6, 27.3)
	Other	Raw	23	1.0 (0.9)	1.4 (1.9)	−0.1 (−0.4, 0.9)
		Rule 1		1.2 (1.0)	1.6 (2.1)	−0.1 (−0.7, 1.0)
		Rule 2		2.0 (2.1)	4.6 (7.0)	0.8 (−0.7, 4.7)
	Uncodable		27	6.1 (6.7)	7.2 (7.9)	0 (−5.0, 6.2)
Weekends	Regulated watching	Raw	22	75.4 (65.9)	86.8 (64.5)	7.3 (−30.3, 60.1)
		Rule 1		80.1 (67.6)	92.9 (69.1)	10.4 (−24.9, 67.1)
		Rule 2		104.2 (77.7)	121.3 (90.4)	34.4 (−44.6, 101.8)
	Unregulated watching	Raw	17	44.9 (57.0)	41.9 (72.7)	−12 (−61.3, 43.6)
		Rule 1		48.3 (62.1)	44.7 (75.6)	−12.6 (−64.1, 46.2)
		Rule 2		70.6 (82.8)	68.3 (86.2)	−13.0 (−92, 58.5)
	Watching unknown	Raw	18	2.8 (3.6)	5.4 (7.4)	0.4 (−2.2, 6.0)
		Rule 1		3.2 (3.8)	5.8 (7.9)	0.2 (−2.8, 5.6)
		Rule 2		6.6 (7.5)	14.8 (32.6)	0.2 (−6.6, 10.3)
	Gaming/watching gaming	Raw	17	70.1 (113.6)	74.6 (118.2)	8.0 (−30.4, 50.1)
		Rule 1		73.3 (118.3)	77.8 (120.8)	8.0 (−28.3, 52.0)
		Rule 2		82.6 (126.1)	100.8 (124.6)	42.3 (−18.5, 89.8)
	Browsing/social media	Raw	22	8.7 (8.7)	8.8 (11.1)	1.4 (−12.6, 5.6)
		Rule 1		9.4 (9.6)	9.4 (12.0)	1.5 (−12.8, 5.8)
		Rule 2		16.6 (18.9)	14.3 (18.6)	1.5 (−9.7, 12.0)
	Communication	Raw	3	0.6 (1.1)	0.0 (0.0)	−0.6 (−0.7, −0.5)
		Rule 1		0.6 (1.1)	0.0 (0.0)	−0.7 (−0.7, −0.5)
		Rule 2		1.1 (0.3)	0.0 (0.0)	−1.0 (−1.4, −0.9)
	Educational/creative	Raw	6	5.0 (7.8)	3.8 (4.0)	1.5 (−12.0, 5.3)
		Rule 1		5.2 (8.1)	4.4 (4.7)	1.8 (−12.6, 6.1)
		Rule 2		5.7 (8.8)	11.6 (18.7)	3.3 (−10.3, 15.4)
	Other	Raw	22	3.8 (6.2)	3.0 (4.3)	0.1 (−1.3, 1.5)
		Rule 1		5.0 (9.1)	3.4 (4.8)	0.5 (−1.5, 1.8)
		Rule 2		8.5 (12.3)	5.8 (7.6)	−0.9 (−4.9, 1.5)
	Uncodable		22	7.7 (7.3)	7.2 (6.7)	0.3 (−5.5, 6.0)

^a^ Before school was the time from when the participant woke up until 9 am on a school day; After school was the time from 3 pm to the time the participant went to sleep on a school day; Weekends was from the time the participant woke up until they went to sleep.

^b^ Regulated watching was defined as any watching that was on a regulated platform (e.g., free to air television, Netflix, Disney+) unregulated watching was defined as any watching that was on an unregulated platform, where members of the public could post videos (e.g., watching video clips on YouTube, Facebook, X (formerly Twitter), TikTok). Social media was defined as interacting with a social media platform, not including watching videos on these platforms (examples of social media include scrolling Instagram, commenting on YouTube videos, posting content on these social media sights). Gaming/watching gaming was defined as playing a video game on any platform, watching someone else play a video game, including on social media websites (e.g., watching a YouTube video of a gamer playing a video game).

^c^ Raw data were calculated from the sum of all images that contained a screen with no assumptions made. Rule 1 included periods where there were no screens as long as this was for 20 s or less (i.e., ≤ 10 images) and there was the same screen time code on either side of the “no screen” episode. Rule 2 included all consecutive images that were coded as blocked or uncodable if there was an image before and after that episode that was coded for the same device and activity (priority screens only).

Mean (SD) parent‐reported screen time during the restriction week 202.2 (114.0) minutes and 185.4 (111.6) minutes during the extension week, leading to a mean (95% CI) difference of 16.8 (1.8, 31.8) minutes between sleep conditions.

## 4 Discussion

Our crossover trial demonstrated that differences in screen time of up to 1 h occur even with relatively low reductions in sleep of just 40 min or so per night. Differences were mostly apparent during the after school and evening periods, with little impact in the before school period or on weekends. In particular, children appeared to watch more television, particularly regulated watching, on school day afternoons when experiencing sleep loss. By contrast, children who engaged in gaming activities tended to game or watch gaming videos more on weekends when sleep was restricted. Such differences in screen use during weekdays might be considered clinically meaningful and provide further experimental evidence of the potential effects of relatively mild levels of sleep loss on health behaviours. However, due to the exploratory nature of these findings, further high‐quality investigation over the long term is needed to confirm the results.

These findings build on the limited existing research that has explored how sleep impacts screen use in children. While cross‐sectional studies frequently report a relationship between sleep and screens, it is mostly assumed that the direction of the relationship is that screens impact sleep [7, 33, 34]. However, interventions that try to improve sleep, including a recent intervention study that removed devices from the family home [35], have had relatively little success at showing that reducing screen use can directly improve sleep outcomes [36]. It is possible that the effect of screen use on sleep may not be as strong as thought, although limitations in measurement and study design may also be affecting these findings [36]. While several longitudinal studies have demonstrated that the relationship is bi‐directional [9, 10], only one previous experimental study appears to have been undertaken. With a similar design to the DREAM study, Hart et al. [12] indicated that parents thought their child had around an extra hour of television time during a sleep restriction protocol. However, this study used a subjective measure of screen use and did not consider the findings in the context of the extra time gained from staying up later. The present study adds novel findings to the existing research, providing objectively measured evidence that sleep loss may increase the amount of time children are spending on screens, even when accounting for the greater time awake.

The biggest differences in total screen use were observed in the after school period, rather than in the weekends or before school. This is potentially because children already have enough time in their day to meet their desired screen use during the weekend. The children were on average using screens on the weekend for around 24%–32% of their day (approximately 3–4.5 h of screen‐use), indicating high screen use on these days. By contrast, the before school period is relatively short and there simply may not be sufficient time for additional screen time to be incorporated due to the constraints of needing to get ready for school. The current results align with previous DREAM findings, which showed that the effects of sleep loss on energy intake appeared to be only on weekdays, presumably because children were already having a high energy intake on the weekends [22]. It is difficult to compare our findings regarding the effect of sleep on screens as studies do not appear to have investigated such effects. For example, Mazzer et al. [10] investigated bi‐directional associations between sleep and screens, but only looked only at weekday screen use, with no data collected at weekends. While Hart et al. [12] collected data on both weekdays and weekends, analyses were not presented separately.

Although total screen use on weekends appeared similar between conditions, there was a tendency for those who engaged in gaming and regulated watching (e.g., Netflix and free‐to‐air television) to engage with these activities more when experiencing sleep loss. Although these analyses are exploratory, they suggest that children who engage in these activities may indeed use them more when given extra time to use them, or when they are tired on the weekends.

There are several potential reasons why children may turn to screens when they sleep less. One likely scenario is that children simply use the extra time awake that is gained from sleeping less to watch screens. However, we specifically tried to control for this additional time by also determining whether the percentage of time allocated to screens differed, observing that children spent 1.6%–6% (depending on the rule used) more time on screens after school as a percentage of their day. These results suggest that these participants chose to use screens more in response to sleep loss, not just as a way of filling in the extra time awake. It is also possible that children used their screens more in that extra time as their parents were not around/awake to restrict their screen use (some children in our study went to bed later than their parents, particularly during sleep restriction). This increase in sedentary time (or at least the screen time component of sedentary time) is supported by previous DREAM analyses that indicated that mild sleep loss led to increases in sedentary time overall [23].

The results from the present study also illustrated how using different rules for coding screen use can impact outcomes in an interventional setting. Mean screen use estimations were substantially lower when coding only screen or no screen (raw data), compared to rule 2 (including consecutive blocked images as screen time if either side of these images contains the same image code). When looking at the median within‐person differences in screen use, using rule 2 changed the estimates of screen use, but tended to still show results in the same direction. For example, the results from the raw coding showed that children only used screens for 18.7 min more within the week of sleep restriction compared to sleep extension; however, when using rule 2, the difference was increased to 66.3 min. These differences in these results change the interpretation of the degree of impact that sleep may have on screen use in children. While other studies have also used assumptions to deal with missing camera data [16, 28, 37] all have done so within their coding protocols, rather than post‐coding as we have done. As such, they are unable to determine the effect of different assumptions, as we have shown here. The strength of our analysis is that as further research comes to light regarding the validity of different rules, we could easily re‐analyse these data without the need to recode, which these previous studies would have to do [16, 28, 37]. We acknowledge that our coding rules are yet to be validated for accuracy, but it is important to note that coding rules can markedly influence screen use estimations in children, and any assumptions (including with missing data) should be clearly outlined in all camera research going forward to allow accurate comparisons between studies.

There were several strengths to this study, including the randomised crossover design with an adequate washout period that allows for a within‐person analysis and thus accounts for both known or unknown confounders. Objective measures of both sleep and screen use were used to estimate changes in sleep and screen use between intervention conditions. In particular, the use of wearable cameras gave estimates for the type of device and screen behaviour. The sample was diverse in terms of weight status, with 40% of the sample classified within the overweight or obese category. However, there were several limitations, including adherence to wearing the cameras. Due to the difficulty of dealing with missing camera data in an interventional context, only those participants who were considered to have high adherence during both weeks were included. This allowed us to assume that missing camera time was not screen use, which could have led to selection bias, with participants who were completing activities where it may be inappropriate to wear cameras (e.g., playing sports) excluded from analyses. Although this may have been the case, it appears that the sample with valid camera data were mainly the participants who were highly adherent to the sleep intervention also, with 78% of the sample meeting the per‐protocol sleep difference compared to 56% of the overall sample. Still, it is important to highlight that due to non‐adherence, this analysis is exploratory, and further research with appropriate sample sizes is needed to confirm these findings. Another limitation was that not all participants had valid data for each time‐block, again limiting the ability to make strong conclusions, while highlighting the difficulties of using wearable cameras to measure screen use in the intervention setting. Although the coding of screen use through wearable camera images was tested for reliability between coders and within coders, this method is yet to be tested for validity (i.e., what we coded was truly what was happening) due to the pragmatic and real‐world setting of this research. Although attempts were made to blind the coders to the intervention order, it was possible that coders were aware of which intervention the participants were completing due to the timestamps on the images. Finally, we did not compare sleep restriction against their habitual sleep because we were uncertain whether children were receiving sufficient sleep to meet their needs at baseline. The fact that two‐thirds of our sample were able to increase their sleep when given the opportunity to do so confirms that many children, even if they meet current sleep guidelines, may not be getting sufficient sleep [38].

In conclusion, this exploratory analysis has added further evidence of the effects of mild sleep deprivation on health. The results suggest that when children get less sleep, they appear to spend more time engaged with screens on weekdays after school, as well as use screens more as a percentage of their day when tired. Further high‐quality research is needed to explore whether improving sleep can reduce screen use in children, while furthering the understanding of how screen time may mediate the relationship between sleep and obesity in children.

## Author Contributions

R.F.J., K.A.M.‐J., J.J.H., B.C.G., S.M. and R.W.T. designed the research. R.F.J., S.M. and M.J. conducted the research. R.F.J. and J.J.H. designed and performed the statistical analyses. R.F.J. had responsibility for the camera data and K.A.M.‐J. for the accelerometry data. R.F.J. wrote the paper. R.W.T. was Principal Investigator and had primary responsibility for the final content. All authors read and approved the final manuscript.

## Conflicts of Interest

R.W.T. is on the editorial board of Paediatric Obesity. The other authors declare no conflicts of interest.

## Data Availability

The data that support the findings of this study are available on request from the corresponding author. The data are not publicly available due to privacy or ethical restrictions.
